# Supplementary material for: Height-diameter allometry for tropical forest in northern Amazonia
Source: PLoS One. 2021 Dec 16;16(12):e0255197. doi: 10.1371/journal.pone.0255197 (PMC8675728; doi:10.1371/journal.pone.0255197)
Supplement: S1 File — Appendix A: Supplementary information for statistical analysis of nonlinear models adjusted to estimate total height in tropical rainforest in Amapá. S1 Fig. Distribution of fixed sampling units of 10 x 10 m to carry out the forest inventory in Area B. Adapted from Oliveira et al. (2012) [46]. Appendix B: Supplementary information for the statistical results of adjustment and selection of non-linear models for total height estimation in tropical rainforest in Amapá. S2 Fig. Predicted vs. scatter plot observed height (m) by the best local model (Weibull). The solid blue line represents the estimated values with gray confidence bands (p < 0.05). Red dashed lines represent predictions for height values. The solid black line represents the 1:1 ratio; S3 Fig. Percent error (validation estimate minus observed Ht, divided by observed Ht, in %) vs estimated height values for the six height prediction alternatives; the thick black line represents a spline regression of the data points. The background represents the density of the data point (n = 54 trees); S1 Table—Comparison of mean height differences generated by local, regional, continental, pan-tropical and hypsometer model estimates with the true mean height measured in the field after tree thinning. Means followed by the same letters did not differ significantly by the Tukey test (p < 0.05). (DOC) [file pone.0255197.s001.doc]

Supplemental information: Height-diameter allometry for tropical forest in northern Amazonia

**Lima et al.**

**Suporting Information I**

**Materials and Methods**

**Study área**

**Fig S1. Distribution of fixed sampling units of 10 x 10 m to carry out the forest inventory in Area B. Adapted from Oliveira et al. (2012).**

**Allometric models**

The goodness of fit of each equation was compared through Akaike Information Criteria (AIC), adjusted coefficient of determination, Root Mean Square Error and bias, as described below:


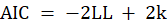
 (4)

where: LL is the log-likelihood and k is the number of parameters of the model. This criterion penalizes the addition of parameters in the analyzed models. Indicates the quality of fit by the equations. The best equation minimizes the value of the AIC.


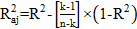
 (5)

where: R2 = coefficient of determination; n = number of observations; k already defined above. By this criterion, the closer to one (1.0) or 100% is the value of the adjusted coefficient of determination, the greater the total variation of the data explained by the independent variables.


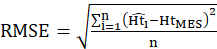
 (6)

Where
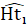
 is the predict total height individual (i);
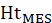
 is the total height measured and *n* is the total number of observations. The lower the RMSE, the better the accuracy.


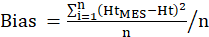
 (7)

This statistic indicates a tendency of under/overestimation, being a measure of error and measure of quality in the realized predictions, so that, the smaller the error, the greater the efficiency in the generalizations.

**Suporting Information II**


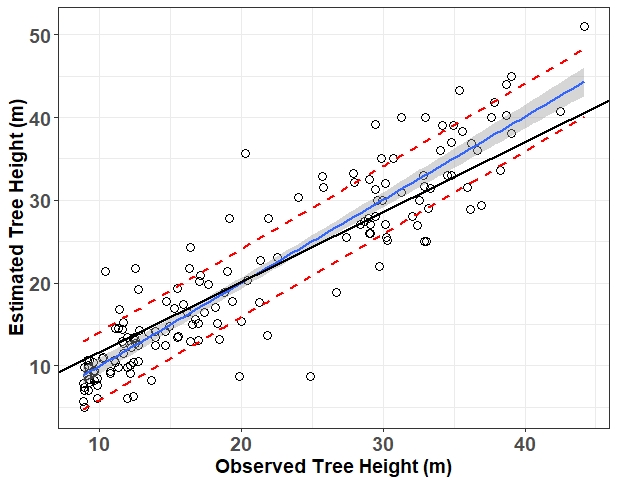


**Fig S2. Percent error (validation estimate minus observed Ht, divided by observed Ht, in %) vs estimated height values for the six height prediction alternatives; the thick black line represents a spline regression of the data points. The background represents the density of the data point (n = 54 trees).**

**
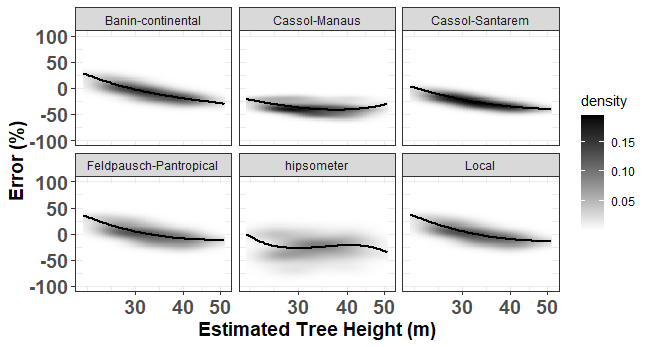
**

**Fig S3. Percent error (estimated Ht minus observed Ht, divided by observed Ht, in%) vs estimated height values for pantropical, continental and regional models; the thick black line represents a spline regression of the data points, illustrating a negative bias as the greatest heights are estimated (values > 30 m). The background represents the density of the data point (n = 54 trees).**

**Table S2 – Comparison of mean height differences generated by local, regional, continental, pan-tropical and hypsometer model estimates with the true mean height measured in the field after tree thinning. Means followed by the same letters did not differ significantly by the Tukey test (p < 0.05).**

| Methods | Mean Height total | Groups |
| --- | --- | --- |
| Feldpausch-Pantropical | 33,22 | a |
| Measured | 33,19 | a |
| Local | 33,11 | a |
| Banin-continental | 30,25 | b |
| Hypsometer | 25,06 | c |
| Cassol-Santarem | 24,32 | c |
| Cassol-Manaus | 20,63 | d |

demo
